# Deep Learning Equalizer Connected with Viterbi-Viterbi Algorithm for PAM D-Band Radio over Fiber Link

**DOI:** 10.3390/s23249773

**Published:** 2023-12-12

**Authors:** Tangyao Xie, Qiang Sheng, Jianguo Yu

**Affiliations:** 1 Beijing Key Laboratory of Work Safety Intelligent Monitoring, Beijing University of Posts and Telecommunications, Beijing 100876, China; xietangyao@bupt.edu.cn (T.X.); yujg@bupt.edu.cn (J.Y.); 2 Key Laboratory of Space Utilization, Technology and Engineering Center for Space Utilization, Chinese Academy of Sciences, Beijing 100094, China

**Keywords:** CVNN equalizer, RVNN equalizer, D-band, PAM, ROF

## Abstract

D-band (110–170 GHz) has been regarded as a potential candidate for the future 6G wireless network because of its large available bandwidth. At present, the lack of electrical amplifiers operating in the high frequency band and the strong nonlinear effect, i.e., the D-band, are still important problems. Therefore, effective methods to mitigate the nonlinear issue resulting from the ROF link are indispensable, among of which machine learning is considered the most effective paradigm to model the nonlinear behavior due to its nonlinear active function and structure. In order to reduce the computation amount and burden, a novel deep learning neural network equalizer connected with typical mathematical frequency offset estimation (FOE) and carrier phase recovery (CPR) algorithms is proposed. We implement D-band 45 Gbaud PAM-4 and 20 Gbaud PAM-8 ROF transmission simulations, and the simulation results show that the real value neural network (RVNN) equalizer connected with the Viterbi-Viterbi algorithm exhibits better compensation ability for nonlinear impairment, especially when dealing with serious inter-symbol interference and nonlinear effects. In our experiment, we employ coherent detection to further improve the receiver sensitivity, so a complex baseband signal after down conversion at the receiver is inherently produced. In this scenario, the complex value neural network (CVNN) and RVNN equalizer connected with the Viterbi-Viterbi algorithm have better BER performance with an error rate lower than the HD-FEC threshold of 3.8 × 10^−3^.

## 1. Introduction

The developing industry could see explosive growth in data traffic once the 5G technology enables various connected services. The high-frequency millimeter wave band above 100 GHz towards the THz band (300 GHz–10 THz) has been presented as an effective candidate to alleviate the problem in the beyond 5G (B5G) and future 6G mobile networks, which have superiority in large bandwidth. It is worth noting that D-band millimeter wave (mm-wave) attracts a great deal of research attention since it has an atmospheric transmission window with a bandwidth of approximately 26 GHz [1]. Photonics-aided approaches have been intensively studied to generate large-capacity mm-wave signals, especially in high-frequency radio over fiber (ROF) transmission, for example, W-band [2], D-band [3], and even THz-band [4]. This method overcomes the bottleneck of electrical devices and is easy to implement for large-capacity mm-wave signal generation.

However, there are also some drawbacks to ROF delivery, one of which is the nonlinear noise resulting from optical fiber and photoelectric devices such as modulators and photodiodes. Lots of efforts have been made to mitigate the nonlinear effect, for instance, equalization in Volterra series, look-up-table pre-distortion, and artificial intelligence (AI), including artificial neural networks (ANN) [5], deep neural networks (DNN) [6], convolutional neural networks (CNN) [7], and recurrent neural network (RNN) models [8]. They have been widely applied in the era of digital signal processing (DSP). In particular, NN frameworks are well established in the equalization process [9], but these real-valued NN equalizers are limited to building a model of complex channels. It implies that a traditional NN equalization model is not suitable for a complex signal inherently produced from QAM modulation format or coherent detection. In Ref. [10], the Volterra equalizer was separately extended to the real and imaginary parts of the QAM signal. The real leaky-*reLU* activate function is also separately applied to both real and imaginary components [11]. However, the complex signals divided into two independent real and imaginary parts ignore their relationship, thus ignoring the potential phase information. Thus, the establishment of a CVNN is essential, as this provides a more constrained system than a real-valued training structure.

Conventionally, NN training is a pure data-driven paradigm in which the optimum identification strategies are decided through network nodes based upon the experience from the previous training set. However, since it reduces the dependence on mathematical models in network design and has a poor mathematical explanation, which usually means it requires plenty of training data and time. In practical wireless applications, with the consideration of transmission efficiency and time delay, the typical mathematical-oriented algorithms and methods should not be completely replaced by the solution of pure data-driven NN. Actually, in our opinion, the joint use of pure data-driven and classical mathematical approaches will greatly benefit a lot for the future high-speed wireless communication. 

In this paper, we extend the analysis by combining a pure data-driven deep learning neural network (DNN) [12,13,14] with typical mathematical models such as frequency offset estimation (FOE) and carrier phase recovery (CPR), which are used to improve the BER decision accuracy and decrease the complexity. We implement the D-band 45 Gbaud PAM-4 and 20 Gbaud PAM-8 ROF transmission simulations. We also experimentally demonstrated 90 Gbit/s/λ D-band PAM-4 D-band signal generation and ROF transmission over 10 km single-mode fiber (SMF) and 3 m free-space wireless transmission. At the receiver, deploying a coherent detection scheme, a pure data-driven DNN equalizer connected with FOE and CPR is employed to simultaneously improve the recovery of the signal. We believe our proposed equalizer has an application prospect for the future 6G mobile network.

## 2. Operation Principle for Deep Learning Equalizer Connected with Viterbi-Viterbi Algorithm

**Figure 1:**
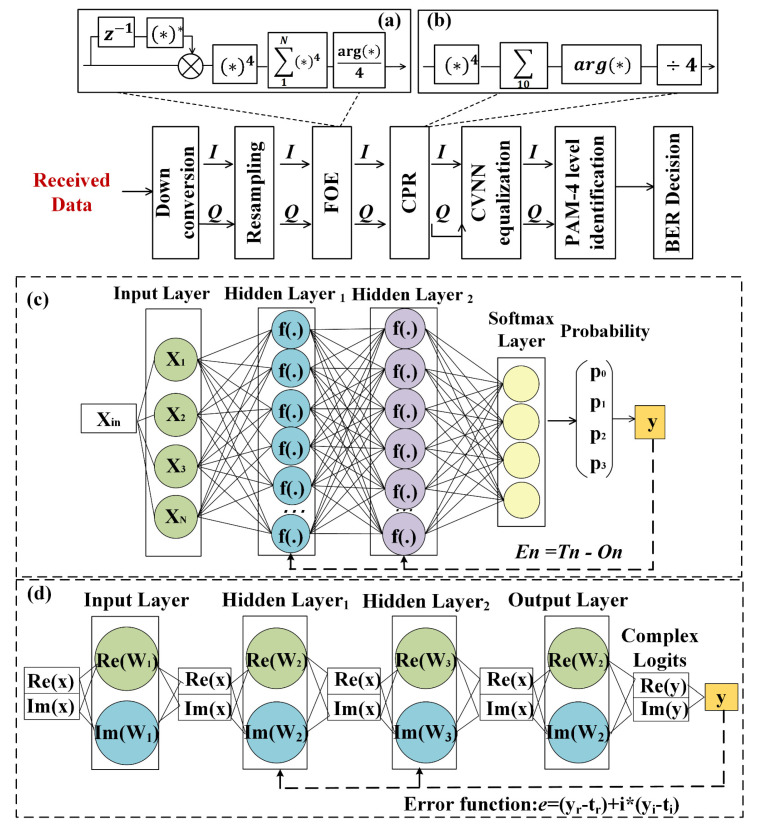
The neural framework of our proposed RVNN and CVNN equalizers connected with Viterbi-Viterbi algorithm, respectively. (**a**) The block diagram of FOE; (**b**) the block diagram of CPR; (**c**) the neural framework of the RVNN equalizer; (**d**) the neural framework of the CVNN equalizer.

Generally, for m-PAM ROF delivery based on the coherent detection scheme, complex intermediate frequency (IF) components occur naturally after down conversion, so there is an inherent connection between the *I* and *Q* parts of the complex numbers. Consequently, as illustrated in Figure 1, the down-converted IF signal is complex-valued and composed of both *I* and *Q* components. Subsequently, FOE and CPR using the classical Viterbi-Viterbi algorithm are deployed to handle the frequency and phase noises. The classical Viterbi-Viterbi algorithm, primarily employed for feedforward carrier recovery, is a prevalent method in optical communication systems for modulation signal recovery [15]. In high-speed optical communication systems, discrepancies in center frequencies between the local oscillator laser and the transmitter can induce rotational and divergent effects on the constellation. This leads to a frequency deviation, impacting the recovery of the received signal. Consequently, the FOE algorithm becomes imperative to offset these frequency deviations. Moreover, laser phase noise introduces phase deviations in the received signals. The CPR approach, based on the Viterbi-Viterbi algorithm, employs the fourth power method to negate the signal’s original phase. Subsequently, phase noise is mitigated by summing and averaging the signal sequences, from which the phase is then estimated by extracting its argument. After the Viterbi-Viterbi algorithm, an RVNN or CVNN equalizer is implemented to mitigate the nonlinear distortion. Finally, the complex PAM pattern is identified into a real four-level distribution, and BER is calculated according to the real PAM-identified signals. 

Figure 1a gives the block diagrams of frequency offset estimation in a coherently detected PAM transmission system. The precondition of the algorithm is that the difference between the modulation phases of the PAM signal is four times a constant phase value. In Figure 1b, the phase offset estimation phase can be obtained by adding and averaging the fourth power operation for the signals after FOE, removing phase noise. As shown in Figure 1c,d, we constructed neural networks including RVNN and CVNN. In particular, the complex training operation in Figure 1d is implemented via the updating of three parameters, including a complex weight value, a complex active function, and complex back-propagation errors.

This study investigates two different equalizer schemes for nonlinear neural networks. The first scheme involves using a RVNN for processing complex baseband signals after digital signal processing algorithms such as FOE and CPR steps in the digital detection system. For N-order modulation signal equalization, N-classification is applied, with multi-classification considered for four-level equalization and classification indices *M* = 4 and *M* = 8 for PAM4 and PAM8 signals, respectively. As illustrated in Figure 1c, a deep neural network (DNN) equalizer with a *softmax* layer as the output layer is employed, consisting of real fully connected layers. The input matrix *X*(*n*) = [*x*(*n*), *x*(*n* − 1)…, *x*(*n* − *N*_0_ + 1)]*^T^* with *N*_0_ memory length is multiplied by the weight value in the hidden layers. Considering the 4-classification modulation signal equalization, the *softmax* function is a generalized logistic function that transforms a real signal sequence with a length of *T* into a probability vector of length 4. To address issues such as the exploding gradient problem, vanishing gradient problem, and convergence rate, the rectified linear unit (*ReLU*) activation function is used due to its linearity and maximum function properties, which have been shown to outperform other activation functions.

(1) ReLU(real)=max(0,real)

The output vectors ${\left[p_{0}{}^{t},\;p_{1}{}^{t},\;p_{2}{}^{t},\;p_{3}{}^{t}\right]}^{T}$ have a sum that is equal to one. The likelihood of the *t*-th symbol is given by this probability distribution:

(2) $$p_{v}^{t}=soft\max(z_{v}^{t})=\frac{\exp(z_{v}^{t})}{\sum_{v\prime =0}^{3}\exp(z_{v\prime }^{t})}(v\in 0,1,2,3)$$

Our study also introduces the complex rectified linear unit (*CReLU*) activation function, described in Equation (3), to a complex deep neural network-based equalizer, which is the second NN equalizer scheme. Prior research on deep learning with complex-valued data has primarily used the *CReLU* activation function for tasks such as channel estimation [16], radar imaging [17], image recognition [18], and voice separation [19]. However, our study proposed to apply the *CReLU* [20] activation function to this particular task innovatively. The approach involves continuously training the complex input through complex neural nodes until convergence of the complex error or completion of a desired number of iterations. As a result, the output value from the linear output layer is also complex. This novel use of the *CReLU* activation function opens up new possibilities for improving the performance of complex deep neural network-based equalizers.

(3) CReLU=ReLU(real)+i⋅ReLU(imaginary)=max(0,real)+i⋅max(0,imaginary)

## 3. Simulation Results and Discussions for PAM-4

**Figure 2:**
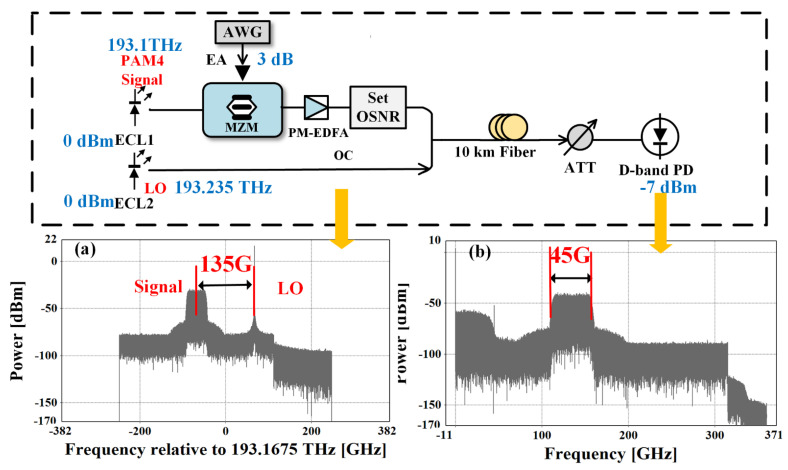
Experimental setup for simulation of D-band PAM-4 radio over fiber transmission at Tx. (**a**) Optical spectra after the optical coupler; (**b**) Electrical spectra of 45 Gbaud PAM-4 signal after PD.

Figure 2 shows the experimental setup for simulating our proposed D-band PAM-4 radio over fiber transmission system at Tx. The PAM-4 signal generated by offline software programming with a length of 2^14^-1 is first DAC converted by AWG with a sampling rate of 80 GSa/s. Amplified by an electrical amplifier (EA), the baseband PAM-4 signal is used to drive an MZM with a 3-dB optical bandwidth of 30-GHz, a half-wave voltage of 2.7-V at 1 GHz, and a 6 dB insertion loss. The external cavity laser (ECL) 1, with a line width of 100 kHz and an average power of 0 dBm, keeps on emitting CW, which is operated at 193.1 THz and carries PAM-4 data. A PM-EDFA is used to boost the transmitted signal after MZM, and the ‘SET OSNR’ module functions to measure the optical SNR in this optical lane. Another ECL2 works at 193.235 THz as a LO to generate D-band mm-wave signals. Figure 2a depicts the corresponding optical spectra of the 45 Gbaud PAM-4 signal after the optical coupler. The coupled light beam is delivered over 10 km of SSMF with a 17-ps/km/nm CD at 1550 nm. An attenuator (ATT) adjusts the optical power to obtain the optimum input power into the adopted photodiode (PD) with an output power of −7 dBm. Since the frequency space between two lasers is 135 GHz, a 135 GHz PAM-4 mm-wave signal is generated based on optical heterodyne beating, and its electrical spectrum is shown in Figure 2b.

**Figure 3:**
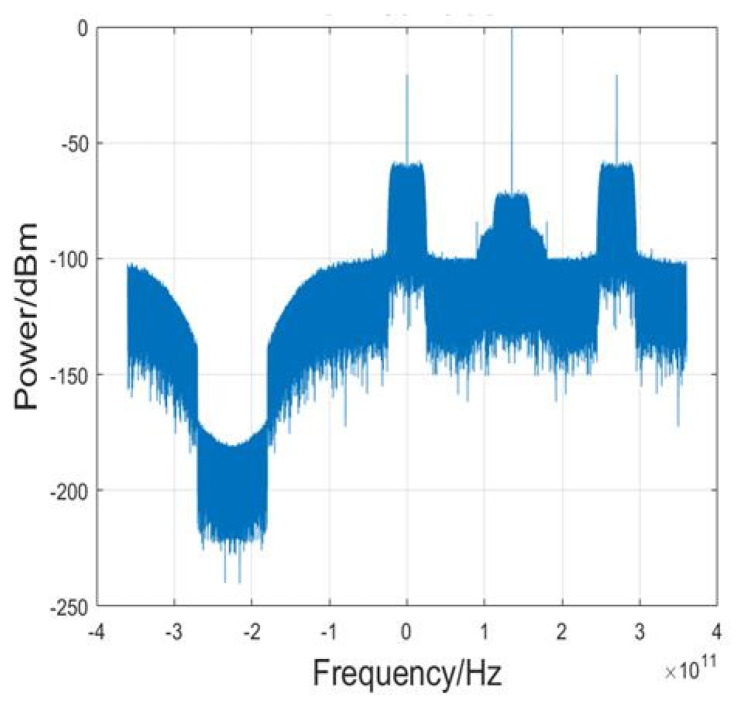
The electrical spectra of baseband 45 Gbaud PAM-4 signal after down conversion.

Figure 3 gives the corresponding electrical spectra of the baseband 45 Gbaud PAM4 signal after down conversion. Except for down conversion, the traditional offline DSP algorithms at Rx include resampling, CMA, FOE, and CPR. To further overcome the nonlinear effect, we employ the RVNN equalization scheme. After repeated tests and comparisons, the neural architecture we’ve adopted is distinctly more parsimonious, characterized by a structure of [371-260-1]. Specifically, this configuration consists of 371 neurons in the input layer, 260 neurons in the hidden layer, and one neuron in the output layer. As substantiated in the ensuing sections, this particular structure efficaciously addresses the demands of nonlinear equalization, eschewing superfluous augmentation of the network’s computational complexity.

### 3.1. BER Performances versus Different SNRs

**Figure 4:**
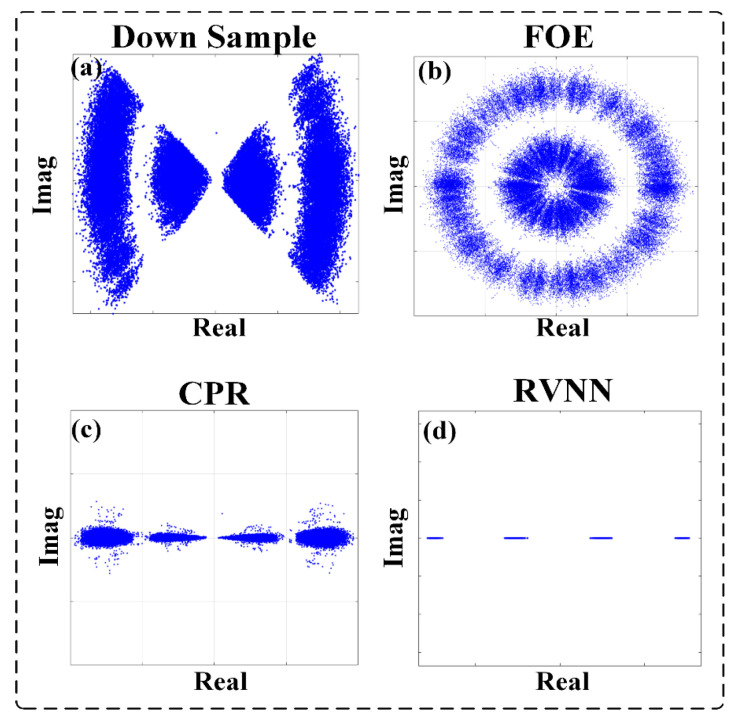
The constellation diagrams of PAM-4 with an OSNR of 45 dB after (**a**) down sample; (**b**) FOE; (**c**) CPR; (**d**) RVNN.

To further test the performance of the RVNN equalizer connected with the Viterbi-Viterbi algorithm scheme and the pure data-driven scheme, we analyze the BER results versus different optical SNR parameters of the transmission system when the line widths of the ECLs are fixed at 100 kHz. The corresponding constellation diagrams for the PAM-4 signal with a high OSNR of 45 dB after down conversion, FOE, CPR, and RVNN equalization are given in Figure 4, respectively. It can be found that when the OSNR is high, for instance, 45 dB, the frequency and phase noise can be completely rectified by the Viterbi-Viterbi algorithm, and the following RVNN equalizers help further improve the BER performance.

**Figure 5:**
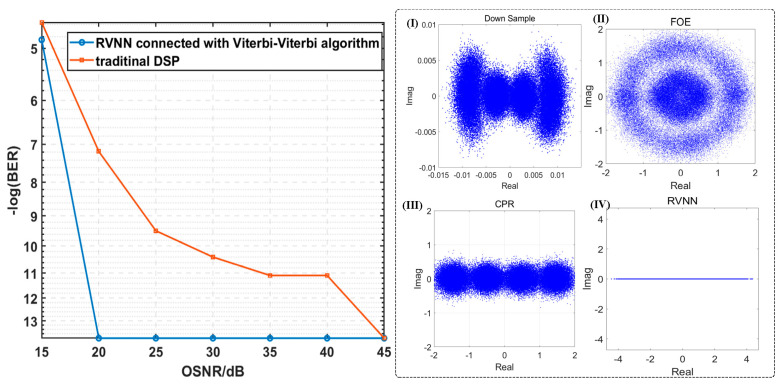
The BER performances of PAM-4 versus different OSNR parameters when the ECLs’ line widths are fixed at 100 kHz, and the corresponding constellation diagrams after insets (**I**) down sample; (**II**) FOE; (**III**) CPR; (**IV**) RVNN when the OSNR is 15 dB.

As seen in Figure 5, the BER performances vary with the OSNR, and for all DSP schemes, the BER drops with the increase in ONSR. Moreover, as seen in the insets (I)–(IV) of Figure 5, the corresponding constellation diagrams for the PAM-4 signal with a low OSNR of 15 dB after different equalization schemes are given, respectively. There is no doubt that the Viterbi-Viterbi algorithms mitigate frequency and phase noise. However, the spaces between the four levels of the PAM-4 signal with a low OSNR are very narrow due to the nonlinear effect. Thanks to the RVNN equalizer, we can observe a further reduction in BER. 

We can draw the conclusion from Figure 5 that as the OSNR increases to 30 dB, the BER decreases to 0, successfully employing any DSP scheme. Moreover, with the aid of Viterbi-Viterbi algorithms, BER is decreased, though it is still high when the OSNR is low. For example, BER is reduced to 0.012 when the OSNR is 15 dB, which is still above the HD-FEC threshold of 3.8 × 10^−3^. Compared with the traditional DSP algorithm, the RVNN equalizer connected with the Viterbi-Viterbi algorithm exhibits better compensation ability for nonlinear impairment, especially when dealing with serious inter-symbol interference and nonlinear effects. It is worth noting that with the increase in OSNR, the BER reduction becomes limited because the transmission channel can meet the PAM-4 signal’s requirements for high SNR, and the inter-symbol interference and nonlinear effects are not significant at this point.

### 3.2. BER Performances versus Different Line Widths of ECLs

**Figure 6:**
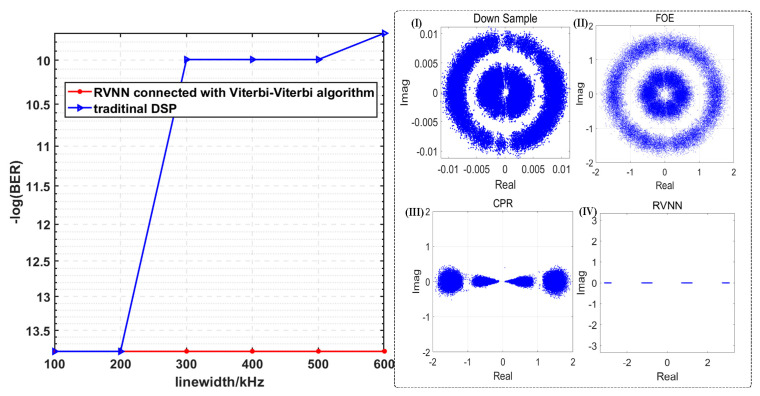
The BER performances of PAM-4 versus different line widths of the ECL when the OSNR is 40 dB and the corresponding constellation diagrams after insets (**I**) down sample; (**II**) FOE; (**III**) CPR; (**IV**) RVNN when the linewidths of the ECL are 600 kHz.

Moreover, we investigate the BER performances employing different DSP schemes versus different line widths of ECL when the OSNR is fixed at 40 dB in Figure 6. And the insets of Figure 6 give the corresponding constellation diagrams after the RVNN equalizer is connected with the Viterbi-Viterbi algorithm, respectively, when the line widths are fixed at 600 kHz. 

It is evident that with the increase in ECLs’ line widths, the BER has increased to 6.05 × 10^−5^. As seen from the constellation diagrams in inset (I), when the line widths of ECL reach 600 kHz, the four constellation points of the PAM-4 signal gradually rotate into two incomplete rings since the phase noise is relatively severe, compared with the instances with a 100 kHz line width. As ECL’s linewidth increases, its relative intensity noise (RIN) increases accordingly, reflecting on the phase noise of the received PAM-4 signals. It can be concluded from the BER results that in an optoelectronic oscillator system, the narrower the laser line width, the better the spectral and phase noise characteristics of the resulting signal. Meanwhile, with the help of RVNN equalizers, the BER performances are significantly improved and can be controlled to 0. It proves the superiority of RVNN equalizers in improving recovery accuracy.

## 4. Simulation Results and Discussions for PAM-8 

**Figure 7:**
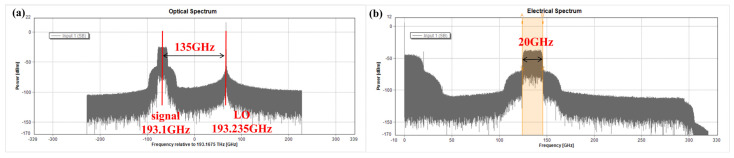
The spectra of the PAM-8 signal. (**a**) The optical spectra after the optical coupler; (**b**) Electrical spectra of 20 Gbaud PAM-8 signal after PD.

We use the same RVNN equalizer with a structure of [371-260-1] as Figure 2 to test the BER performance of 20 Gbaud PAM-8 signals. Figure 7a,b give the corresponding optical spectra after the optical coupler and the electrical spectra of the 20 Gbaud PAM-8 signal after PD for simulation, respectively. 

**Figure 8:**
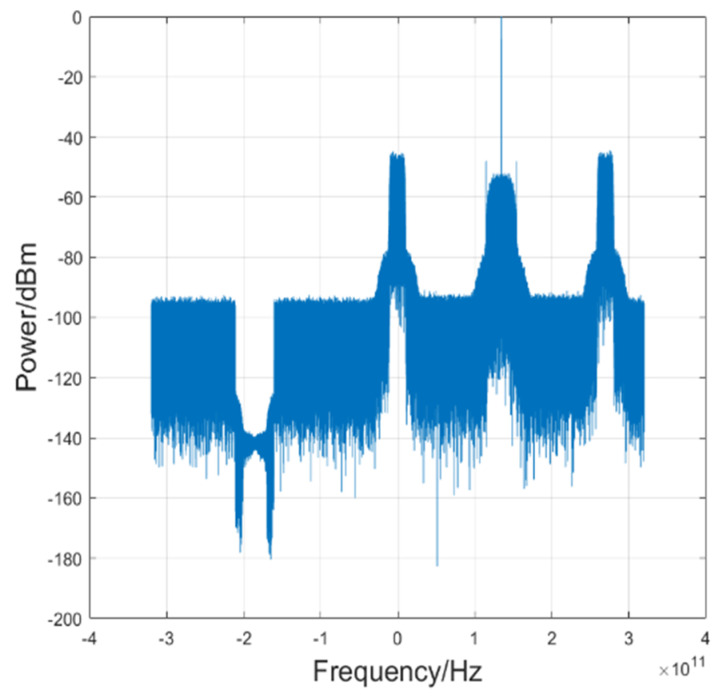
The electrical spectra of baseband 20 Gbaud PAM-8 signal after down conversion.

On the Rx-side, similar to PAM-4 signals, the DSP process includes down conversion, resampling, the Viterbi-Viterbi algorithm, and the real DNN. After down conversion, the baseband electrical spectra of the 20 Gbaud PAM-8 signal are shown in Figure 8.

### 4.1. BER Performances versus Different SNRs

**Figure 9:**
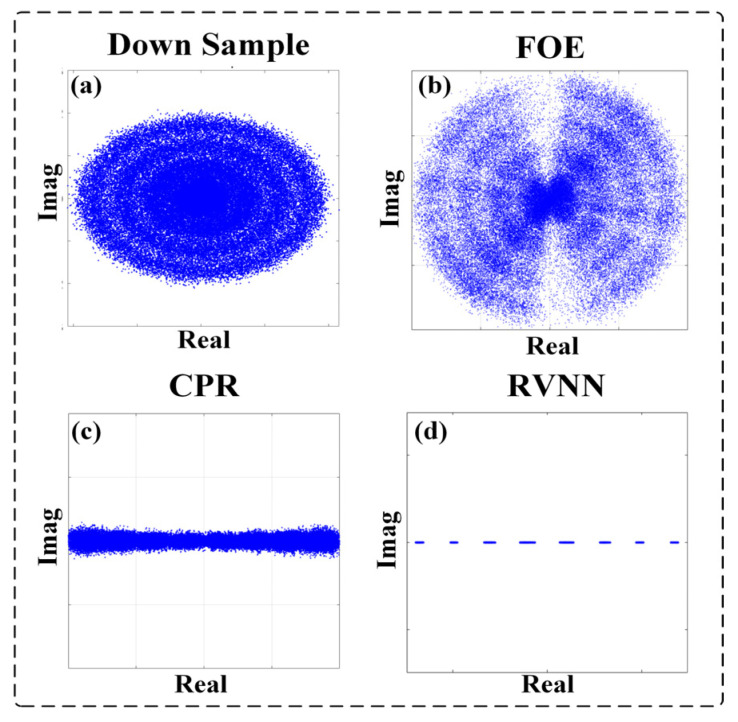
The corresponding constellation diagrams of PAM-8 with an OSNR of 40 dB after real DNN equalizer connected with Viterbi-Viterbi algorithm.

Similarly, we analyze the BER results versus different optical SNR parameters of the transmission system when the line widths of the ECLs are fixed at 100 kHz. The corresponding constellation diagrams for the PAM-8 signal with a high OSNR of 40 dB after down conversion, FOE, CPR, and RVNN equalization are given in Figure 9, respectively. It is evident that when the OSNR is as high as 40 dB, the frequency and phase noise can be recovered by the Viterbi-Viterbi algorithm, while the eight levels of PAM-8 signals cannot be separated from each other due to the nonlinear effect. Thanks to the RVNN equalizers, the BER significantly drops to 0, with completely separated constellation diagrams. 

**Figure 10:**
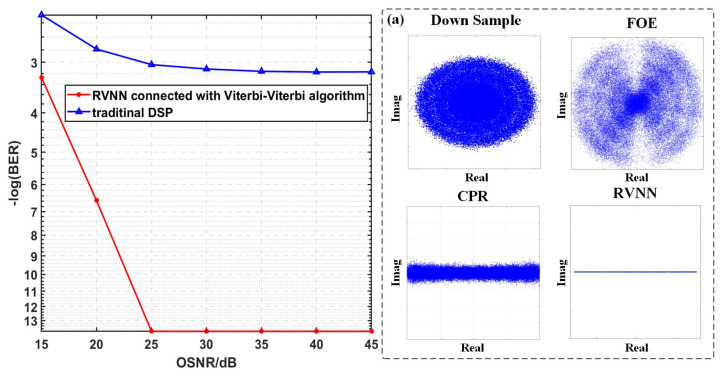
The BER performances of PAM-8 versus different OSNR parameters when the ECLs’ line widths are fixed at 100 kHz, and the corresponding constellation diagrams after (**a**) real DNN equalizer connected with Viterbi-Viterbi algorithm when the OSNR is 20 dB.

Figure 10 gives the BER performances for PAM-8 signals versus the OSNR, and it is obvious that the BER drops with the increase in ONSR. Moreover, Figure 10a describes the corresponding constellation diagrams for the PAM-8 signal after an RVNN equalizer connected with Viterbi-Viterbi algorithms. Compared with the constellation diagrams of PAM-8 signals after down sampling in Figure 9, it can be found that the spaces between the four circles in Figure 10a are squeezed and become narrower, proving that the nonlinear effect increases with the decrease of ONSR. Therefore, the real part of PAM-8 signals cannot be totally classified after employing the RVNN equalizer. Nevertheless, the RVNN can counteract the nonlinear effects well, even at very low OSNR. We can draw from Figure 10 that there is no doubt that RVNN equalizers combined with the Viterbi-Viterbi algorithm made a huge contribution to the decrease in BER.

### 4.2. BER Performances versus Different Line Widths of ECLs

**Figure 11:**
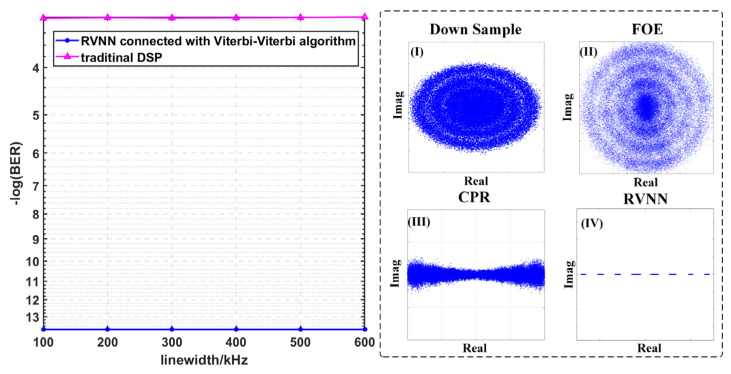
The BER performances of PAM-8 versus different line widths of the ECL when the OSNR is 40 dB, and the corresponding constellation diagrams after insets (**I**) down sample; (**II**) FOE; (**III**) CPR; (**IV**) RVNN when the linewidths of the ECL are 600 kHz.

Similarly, we investigate the BER performances of PAM-8 employing different DSP schemes versus various line widths of ECL when the OSNR is fixed at 40 dB in Figure 11. And the insets of Figure 11 give the corresponding constellation diagrams after the RVNN equalizer is connected with the Viterbi-Viterbi algorithm, respectively, when the line widths are fixed at 600 kHz. Compared with the BER curve of PAM-4, here, we cannot see a significant BER increase for PAM-8 with the increase in line widths of ECLs. Overall, the BER is slowly increasing. When comparing the insets in Figure 11 with Figure 9, we can draw the conclusion that with the increase of ECLs’ line widths, there is a phase offset happening to the constellation diagrams of PAM-8 signals, and the phenomenon can be seen vividly through the diagrams after FOE. Thanks to the CPR algorithm based on the Viterbi-Viterbi model, such phase rotation can be mitigated in most cases, which hints at the reason why the increase in BER is not evident when the ECL’s line widths grow over a range. Meanwhile, with the aid of RVNN equalizers, we can observe a considerable BER drop, which can be controlled to 0. It further proves the superiority of RVNN equalizers in improving recovery accuracy.

## 5. Experimental Setup

**Figure 12:**
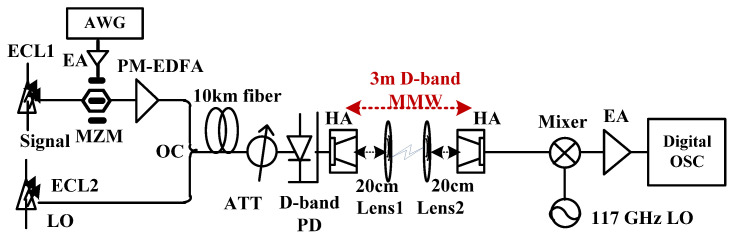
Experimental setup of D-band PAM-4 radio over fiber transmission over 10 km SMF and 3 m free space wireless distance.

Figure 12 shows the experimental setup of our demonstrated 135 GHz PAM-4 mm-wave ROF link over 10 km SSMF and 3 m free space wireless distance, where the transmitted PAM-4 signal distribution X(k) ∈ [−3, −1, 1, 3]. At the transmitter, X(k) is offline processed via PAM-4 mapping and resampling steps. The produced baseband PAM-4 signal produced by offline Matlab software is first digital-to-analog converted (DAC) via AWG with a sampling rate of 80 GSa/s.

**Figure 13:**
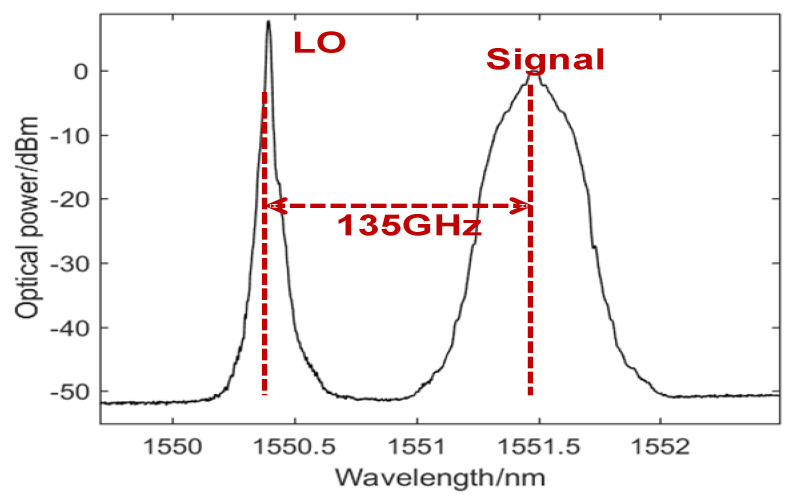
Optical spectra after the optical coupler.

Then the intensity modulator MZM is driven by the baseband PAM-4 signals boosted from an electrical amplifier (EA), which has a 3 dB optical bandwidth of 30 GHz, a half-wave voltage of 2.7-V at 1 GHz, and a 5-dB insertion loss. The output light wave from ECL 1 with a line width of 100 KHz and an average power of 16 dBm is modulated by MZM to carry the modulated PAM-4 data, which is subsequently combined with ECL2 working at 1550.26 nm as a LO. Therefore, the frequency space between ECL1 and ECL2 is 135 GHz. Figure 13 shows the corresponding optical spectra (0.01 nm resolution) after the optical coupler, and it is clear that ECL1 operated as a signal light carries 45 Gbaud PAM-4 data.

After transmission over 10 km SSMF with 17-ps/km/nm CD at 1550 nm, the optical power of the coupled light is adjusted by the attenuator (ATT), then heterodyne beat by the cascaded photodiode (PD) within the frequency range of 10~170 GHz at −2 V DC bias and an output power of −7 dBm. Thus, a 135 GHz mm-wave signal carrying PAM-4 information is generated. Due to the large atmospheric loss at D-band, a pair of identical D-band horn antennas (HAs) with a gain of 25 dBi gain and are deployed to transmit and receive a 135 GHz signal. Moreover, a pair of lenses (Lens 1 and Lens 2) located between HAs are essential to further amplify the 135 GHz PAM-4 signal, the diameter and focal length of which are 10 and 20 cm, respectively. At the receiver, the received 135 GHz signal is first downconverted into an IF signal by a commercial D-band mixer with a 9.5 dB conversion loss and a LO operated at 112 GHz. Therefore, a 23 GHz IF signal is downconverted and then amplified via an EA with a gain of 33 dB and a saturation output power of 14 dBm within the frequency band from DC to 50 GHz. Finally, the signal is captured by a digital storage oscilloscope (OSC) with a sampling rate of 120 GSa/s and a 3-dB electrical bandwidth of 45 GHz.

For DSP steps implemented via offline Matlab software, downconversion into baseband electrical signals and resampling are inevitable. Typical FOE and CPR models are applied before CVNN equalization, and finally the BER decision. 

## 6. Experimental Results

**Figure 14:**
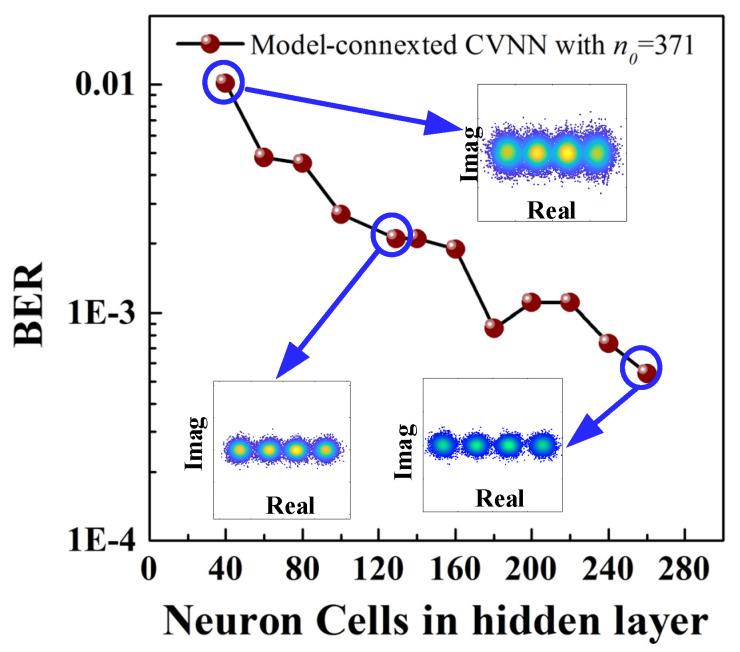
BER performance vs. the neurons in hidden layer for 45 Gbaud PAM-4 signal wireless transmission by employing model-connected CVNN equalizers.

We investigate the influence of neural cells *n*_1_ in the hidden layer of CVNN [371-*n*_1_-1] on BER performance. Here, 371 is the number of neural cells in the input layer, *n*_1_ defines that in the hidden layer, and one represents that in the output layer. According to our test, the Adam Optimizer is used, the batch size is 128, and the network requires 20 epochs to converge. As shown in Figure 14, it can be found that BER decreases with the increasing neuron nodes. For example, BER is as large as 1 × 10^−2^ when the number of neural cells is only 40. Conversely, BER is reduced to 5 × 10^−4^, which corresponds to the scenario when 260 neural units are employed in the hidden layer. In this scenario, the PAM-4 constellation diagram is averagely distributed and clear, while the constellation is fuzzy with only 40 neurons.

**Figure 15:**
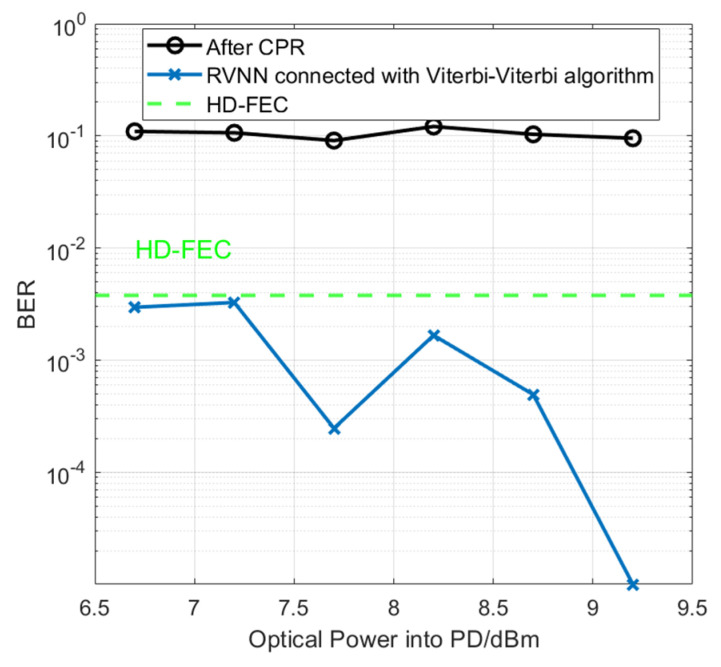
BER performance vs. the optical power into PD for 45 Gbaud PAM-4 signal wireless transmission between traditional DSP algorithms after FOE and CPR and RVNN equalizer [371-260-1] connected with the Viterbi-Viterbi algorithm, respectively.

As we know, several kinds of equalizers are applied in wireless communication, including linear CMA equalizers, linear DD-LMS equalizers, nonlinear Volterra equalizers, and deep learning equalizers. In general, nonlinear equalizers outperform linear ones in terms of higher decision accuracy and fewer minor residual errors. In the experiment, we compared traditional DSP equalizers after FOE and CPR steps with our proposed RVNN equalizers [371-260-1] connected with the Viterbi-Viterbi algorithm. Figure 15 shows the BER performance of 45 Gbaud PAM-4 at 135 GHz versus the optical power into PD. It can be concluded that the traditional DSP algorithm is not very robust against nonlinear noise compared with NN approaches. RVNN equalizer [371-260-1] connected with the Viterbi-Viterbi algorithm has a better BER performance with an error rate lower than the HD-FEC threshold of 3.8 × 10^−3^, among all optical power ranges. Thanks to the help of the traditional Viterbi-Viterbi algorithm, the signal after FOE and CPR is more easily demodulated since frequency and phase noises are mitigated. When the optical power in PD is 9.2 dBm, error-free operation can be achieved.

## 7. Conclusions

In this paper, we propose a novel deep learning equalization scheme connected with the traditional mathematical-oriented Viterbi-Viterbi algorithm. We implement D-band 45 Gbaud PAM-4 and 20 Gbaud PAM-8 ROF transmission simulations, respectively. The simulation results show that the RVNN equalizer connected with the Viterbi-Viterbi algorithm exhibits better compensation ability for nonlinear impairment, especially when dealing with serious inter-symbol interference and nonlinear effects. We also experimentally demonstrated 90 Gbit/s/λ D-band PAM-4 D-band signal generation and ROF transmission over 10 km single-mode fiber (SMF) and 3 m free-space wireless transmission. The experimental results show that, thanks to the help of the traditional Viterbi-Viterbi algorithm, the signal after FOE and CPR is more easily demodulated since frequency and phase noises are mitigated. When the optical power into PD is 9.2 dBm, error-free operation can be achieved by using deep learning neural network equalizers. Therefore, DNN, including RVNN and CVNN, is expected to address nonlinear equalization in optical communications within the context of future 6G systems. By learning the mapping relationship of the non-linear transmission channel, DNN can improve signal quality, exhibit adaptability, and potentially reduce costs. This improvement is critical for achieving long-distance, high-capacity optical communications in 6G systems. Therefore, we envision future 6G wireless networks where traditional mathematical-oriented algorithms and AI-based DSP techniques are implemented in synergy. 

## Data Availability

Data are contained within the article.
